# Design and development of a portable low-cost QCM-based system for liquid biosensing

**DOI:** 10.1007/s10544-024-00696-0

**Published:** 2024-01-18

**Authors:** Mohamed Adel, Ahmed Allam, Ashraf E. Sayour, Hani F. Ragai, Shinjiro Umezu, Ahmed M. R. Fath El-Bab

**Affiliations:** 1 Department of Mechatronics and Robotics Engineering, Egypt-Japan University of Science and Technology (E-JUST), Alexandria, 21934 Egypt; 2 Mechanical Engineering Department, Helwan University, Cairo, 11792 Egypt; 3 Department of Electronics and Communications Engineering, Egypt-Japan University of Science and Technology (E-JUST), Alexandria, 21934 Egypt; 4 Molecular Biomimetics Research Group, Animal Health Research Institute, Agricultural Research Center, Giza, 12618 Egypt; 5 Electronics and Communications Department, Faculty of Engineering, Ain Shams University, Cairo, 11517 Egypt; 6 Department of Modern Mechanical Engineering, Waseda University, 3-4-1 Okubo, Shinjuku-Ku, Tokyo, 169-8555 Japan

**Keywords:** QCM, Flow cell, Hartley oscillator, Peltier module, Micropump

## Abstract

Quartz crystal microbalance (QCM) is a versatile sensing platform that has gained increasing attention for its use in bioapplications due to its high sensitivity, real-time measurement capabilities, and label-free detection. This article presents a portable QCM system for liquid biosensing that uses a modified Hartley oscillator to drive 14 mm-diameter commercial QCM sensors. The system is designed to be low-cost, easy to use, and highly sensitive, making it ideal for various bioapplications. A new flow cell design to deliver samples to the surface of the sensor has been designed, fabricated, and tested. For portability and miniaturization purposes, a micropump-based pumping system is used in the current system. The system has a built-in temperature controller allowing for accurate frequency measurements. In addition, the system can be used in benchtop mode. The capability of the present system to be used in liquid biosensing is demonstrated through an experimental test for sensitivity to changes in the viscosity of glycerol samples. It was found to have a sensitivity of 263.51 Hz/mPa.s using a 10 MHz QCM sensor. Future work regarding potential applications was suggested.

**Graphical Abstract:**

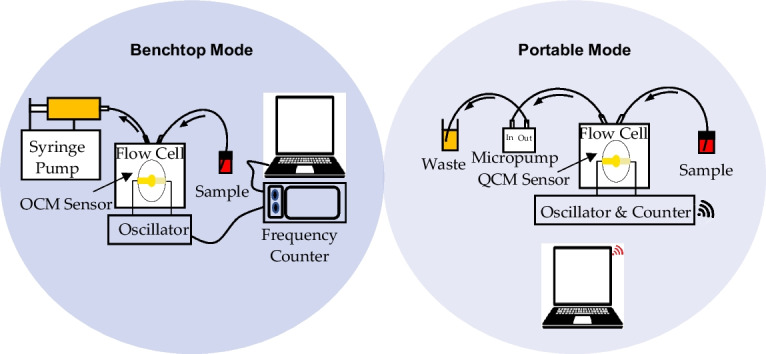

## Introduction

Quartz crystal microbalance (QCM) is a highly sensitive technique that has been widely used in various fields, including biosensors. QCM biosensors have been used in a diverse set of bioapplications, including but not limited to disease diagnosis, drug discovery, environmental monitoring, and food safety. For instance, QCM biosensors have been used for the detection of cancer biomarkers, such as prostate-specific antigen (PSA) and carcinoembryonic antigen (CEA), in serum samples with high sensitivity and specificity (Jandas et al. 2020; Pohanka 2021). In drug discovery, QCM biosensors have been used to monitor the real-time interactions between drugs and their targets, such as enzymes and receptors (Tetyana et al. 2021). Additionally, QCM biosensors have been used for environmental monitoring, such as detecting heavy metals and pesticides in water (Erbahar et al. 2012; Sartore et al. 2011), and for food safety, such as detecting foodborne pathogens (Fulgione et al. 2018; Vaughan et al. 2003). Notably, QCM excels in both liquid and gas sensing realms, as evidenced by recent works in liquid sensing (Khoirudin et al. 2023; Lino et al. 2023) and gas sensing (Aflaha et al. 2023a, b, c; Debabhuti et al. 2022). These research works underscore the versatility of QCM technology in meeting a spectrum of sensing application needs.

The development of portable QCM systems is essential due to the increasing need for on-site and in-field monitoring applications. Portable QCM systems provide the added benefits of reduced instrument size and cost.

Park et al. (2022) presented a miniaturized QCM measurement system as an alternative to benchtop instruments. The use of QCM as part of a portable system for measuring fine particles has been reported (Lee and Kim 2022). Different portable gas-sensing systems based on QCM technology have been proposed, including some utilizing a serial communication interface with a computer (Debabhuti et al. 2022). In contrast, others employ a microcontroller instead of a computer (Banerjee et al. 2020), and some even allow for monitoring through web browsers (Suzuki et al. 2020). These systems consist mainly of QCM sensors, oscillating circuits, and microcontroller-based frequency counters. Liang et al. developed a portable QCM system with a three-layer flow cell for biosensor applications (Liang et al. 2016). However, the need for a benchtop frequency counter limits the functionality of the portable system. In this work, we aim to address the shortcomings of existing portable QCM systems, such as the absence of a built-in temperature controller. Enhancing the system with features like a micropump-based sample pumping system makes it more suitable for use in liquid biosensing.

The QCM technique involves measuring the change in frequency of a quartz crystal resonator that occurs when a mass is added or removed from its surface. The detection of changes in frequency is typically achieved using three electronic techniques: impedance-based measurements, ring-down analysis method, commonly called quartz crystal microbalance with dissipation monitoring (QCM-D), and oscillator-based measurements (Park and Choi 2020). Impedance measurement and QCM-D systems are heavily reliant on software, and due to their high cost and bulky size, they are not suitable for portable systems (Mills et al. 2006; Park et al. 2022; Wudy et al. 2008). Oscillator-based QCM systems are commonly used to characterize gas and liquid phase applications. Additionally, their low cost, compact circuitry, and ability to be customized to fit various applications make them a popular choice for studying the properties of deposited films and liquids (Alassi et al. 2017).

Researchers initially adopted electronic oscillator circuits commonly used as LC tank oscillators (Barnes 1991). The Colpitts, Pierce, and Hartley oscillators are classic examples of electronic oscillator circuits. When designing electronic oscillators in bioapplications QCM systems, it is crucial to consider the specific application and operating conditions, particularly for highly damped mediums. For example, it is recommended to use an oscillator design that grounds one side of the crystal (Barnes 1991, 1992). Colpitts and Pierce oscillators have some limitations under damped conditions (Alassi et al. 2017). The Colpitts oscillator has high gain sensitivity to resonator losses, while the Pierce oscillator lacks grounding on one of its crystal electrodes (Barnes 1991). An amplifying stage has been proposed to improve the oscillator’s stability (Koçum et al. 2009). However, this addition increases the circuit complexity (Alassi et al. 2017).

The Hartley oscillator has several advantages over other oscillators for in-liquid applications, including low cost, stability, and simplicity. Additionally, one of its crystal electrodes is inherently grounded. The Miller oscillator, a modified version of the Hartley oscillator, has gained the attention of several researchers due to its ability to function effectively under different operating conditions (García-Martinez et al. 2011; Rodriguez-Pardo et al. 2007, 2008). Miller oscillators have been found to maintain enhanced noise performance with comparable frequency stability levels, and sustain oscillation under higher damping conditions (Rodriguez-Pardo et al. 2011). In the current work, a modified Hartley oscillator is designed and used in the developed system.

Flow cells play a vital role in QCM systems designed for bioapplications. These cells facilitate regulating the flow of biological samples across the surface of the QCM sensor. A crucial factor to remember while selecting materials for QCM flow cells is their biocompatibility. Another important consideration is the cost of materials and fabrication. Compared to polydimethylsiloxane (PDMS), glass, quartz, and silicon flow systems are more expensive and require complex fabrication techniques, such as photolithography and etching (Chang et al. 2003). The standard soft lithography methodology has been used to fabricate PDMS microfluidic systems. However, the need for a clean room environment and expensive equipment increases the fabrication cost and time (Chiarello et al. 2017; Faustino et al. 2016; Greener et al. 2010). Due to its availability, low cost, biocompatibility, and transparency, PMMA material was used to fabricate microfluidic systems in different bioapplications, such as cell separation, PCR, and DNA systems (Adel et al. 2023; Islam et al. 2018; Nasser et al. 2019, 2021).

Introducing microfluidic systems has significantly improved QCM flow cells for bioapplications. Michalzik et al. (2005) fabricated a flow cell for immunosensor applications using PDMS. The flow cell was permanently bonded with the QCM using the oxygen plasma surface treatment bonding procedure. Similarly, Thies et al. (2018) developed an embedded flow cell from PDMS with a microfabricated QCM device for measuring biological samples utilizing particles. Calero et al. (2020) proposed a multichannel microfluidic cell for bioanalytical applications of monolithic QCM consisting of two parts: a flexible gasket from PDMS and the body of the cell from PMMA. A flow cell for holding high fundamental frequency QCM sensors has been presented by Sagmeister et al. (2009). The main structure of the flow cell was made of aluminium, while PDMS was used as a sealant. In this work, we fabricate the flow cell from the PMMA material through direct writing with a CO_2_ laser beam. This fabrication technique eliminates the need for a photomask and a clean room environment, reducing the fabrication time and the cost of the flow cell.

Temperature stability is crucial in achieving accurate and precise measurements in QCM systems (Magni et al. 2022). Temperature changes affect the frequency and quality factor of the QCM, leading to errors in sensing results. Different types of temperature controllers have been developed to maintain the temperature stability of QCM sensors. One approach is to use a thermoelectric cooler (TEC), also known as a Peltier, to control the temperature of the QCM (Koçum et al. 2009). Li et al. (2018) proposed a QCM dew point sensor structure with temperature control, utilizing two copper wire electrodes bonded on both sides of the QCM sensor using conductive adhesives. In numerous integrated temperature control systems, thermal actuators are positioned close to or in contact with the electrodes of the QCM sensor. These arrangements ensure adequate temperature control but can expose the QCM to local electromagnetic interference emissions from the actuator (Jang et al. 2022; Zampetti et al. 2017).

A considerable amount of recent research has been conducted using QCM sensors across a broad range of temperatures (Nowocień et al. 2022). Wachiralurpan et al. (2021) developed a DNA hybridization biosensor using QCM operating at 60 °C. Huellemeier et al. (2022) researched the adsorption and fouling of milk fractions on stainless steel at 25, 50, and 65 °C. Therefore, the temperature of the oscillating circuit and the QCM sensor may need to be controlled separately. Nowocien et al. (2022) developed a temperature control system using TEC modules, employing two independent temperature regulators. The first is to control the temperature of the QCM sensor to the desired level, and the second is to maintain the temperature of the oscillating circuit at a constant value. The current work uses a single Peltier module to build the integrated temperature control system for miniaturization and cost-reduction purposes.

This work presents a QCM-based system developed to meet high sensitivity requirements, using a modified Hartley oscillator circuit, allowing the detection of minimal mass changes over the sensor surface. The flow cell of the system is developed from PMMA as biocompatible low-cost material. The built-in temperature controller, the piezoelectric micropump, and the Wi-Fi communication module support the portability of the system for field applications. The capability of the presented system to be used in liquid biosensing is demonstrated by testing it on sensing the viscosity of different glycerol samples. The present article is organized as follows. The used material and applied methods for the developed system design, fabrication, and testing are presented in Section 2. Then, Section 3 reveals and discusses the results of the performed experimental work for the system fabrication and the sensitivity validation test. Finally, conclusions are reported in Section 4.

## Materials and methods

### System description

**Fig. 1:**
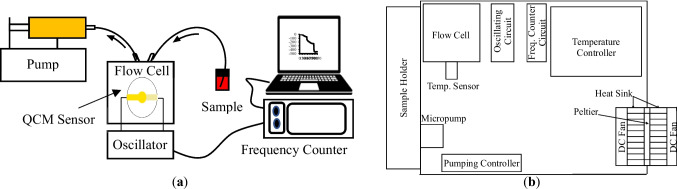
**a** Schematic diagram of in-liquid QCM-based system. **b** Layout of the proposed system

A QCM sensor typically consists of a thin quartz crystal mounted between two metal electrodes, typically made of gold, used to apply an alternating current (AC) to the crystal. When an AC is applied to the electrodes, the quartz crystal oscillates at a resonant frequency. Once a material contacts the surface of the crystal, it causes a change in the mass or viscosity over the sensor surface, which alters its resonant frequency. Continuously monitoring these frequency changes over time allows for detecting and quantifying the mass or viscosity of the material. In an oscillator-based QCM system, the sensor is driven by an oscillating circuit that connects to a frequency counter and a computer for data logging and analysis. The system also includes a flow cell to introduce the material and control its flow over the sensor surface through a pumping system for in-liquid applications. Fig. 1a presents a schematic diagram of a QCM-based system.

### System design considerations

The developed system was designed to work in both benchtop and portable modes. Achieving that requires careful consideration of several factors. Firstly, the oscillator circuit must be designed to achieve high-frequency stability and low-phase noise under conditions of both modes. Secondly, the flow cell design should be compatible with the available commercial QCM sensors to facilitate the sample delivery to the sensing surface while minimizing the effects of fluid dynamics. Thirdly, the pumping system must be designed to provide controlled fluid flow in both modes, considering flow rate and pressure factors. Finally, the temperature controller must be designed to maintain a constant temperature of the quartz crystal in both modes, with consideration given to power consumption in the portable mode. These considerations provide accurate, reliable, and reproducible measurements for various bioapplications in both benchtop and portable modes. Therefore, the developed system consists mainly of a flow cell that holds the QCM sensor, a modified Hartley oscillator circuit connected to a frequency counter circuit, and a micropump-based pumping system. The system also includes an air conditioning system based on a Peltier module. The layout of the developed system is shown in Fig. 1b, and the detailed design and fabrication of each sub-system are described in the following sections.

### Flow cell

**Fig. 2:**
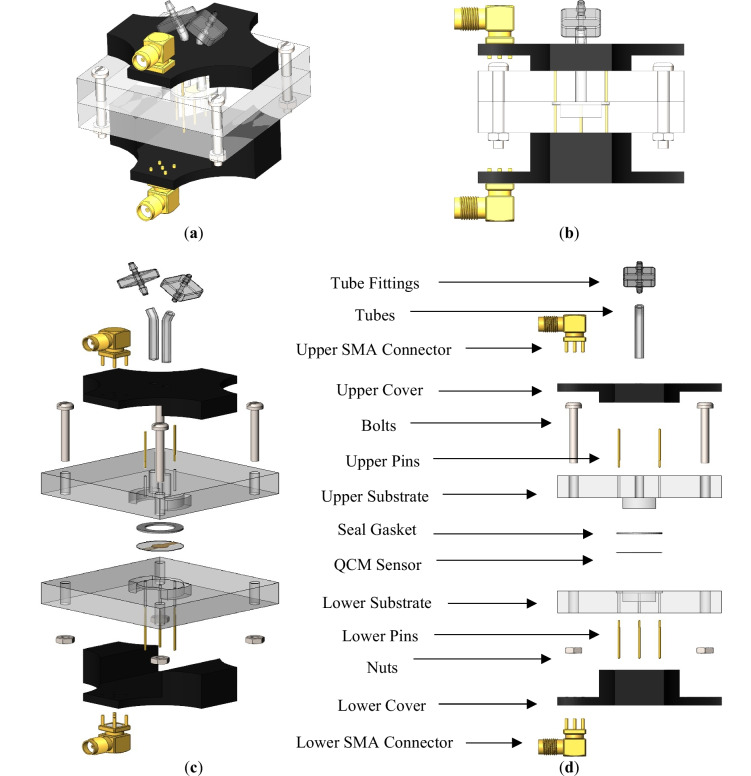
3D design of the developed flow cell. Assembly drawing: **a** isometric and **b** side views. Disassembly drawing: **c** isometric and **d** side views

The main purpose of a flow cell in a QCM system is to support the QCM sensor mechanically and allow delivering the sample solution to the surface of the sensor without leakage (Sagmeister et al. 2009). It also allows for electrical contact between the sensor electrodes and the oscillating circuit. The current work aimed to create a flow cell with a user-friendly design and a QCM sensor that can be replaced easily. The flow cell can also be cleaned using standard laboratory procedures. The flow cell was designed using SolidWorks software. Fig. 2 illustrates the parts of the developed flow cell.

The main structure of the developed flow cell consists of four PMMA layers: two substrates and two covers. The upper and lower covers were fixed and glued to the upper and lower substrates. The dimensions of each substrate are 50 × 50 × 8 mm. Two PMMA parts were fixed in the lower surface of the upper substrate to work as side boundaries of a chamber holding the QCM sensor. The two side boundaries fit into corresponding slots grooved in the lower substrate for tightening and alignment. The input and output ports of the chamber were drilled into the upper cover and the upper substrate, respectively. Then, 1.6 mm-inner diameter connection tubes were fixed and connected to the ports from one side and plastic barbed tube fittings from the other to create a tubing system that connects the liquid reservoir and the QCM sensor. This system allows for delivering the sample solution to the sensor surface. A flexible seal gasket from biocompatible silicon rubber was placed between the upper substrate and the upper surface of the sensor, forming a flow channel. The gasket prevents leakage and ensures uniform fluid flow. The chamber was designed to hold 14-mm-diameter QCM sensors. Additionally, the flow channel has a volume of 50 µL.

To connect the QCM sensor to the oscillating circuit, spring pins are fixed in the substrates and the covers, respectively. Five pins are fixed in the lower part of the cell and two pins in the upper part to make balance over the surface of the sensor and avoid damaging the sensor while assembling the parts of the cells. Many lower pins force the sensor to be in contact with the gasket and ensure contact with the electrodes of different commercial QCM sensors with different electrode shapes. The upper and lower spring pins are connected to Sub-Miniature Version A (SMA) connectors fixed on the edges of the upper and lower covers. These connectors allow for the connection of the QCM sensor to the oscillating circuit through SMA cables. Slots were designed in the middle of each cover to enable faster temperature regulation via the air conditioner. The lower slot also mounts a temperature sensor for feedback signals. After properly mounting the QCM sensor, the upper and lower substrates are assembled using four bolts and nuts.

The flow cell was fabricated using a CO_2_ laser machine (VLS3.5, Universal Laser Systems, Kanagawa, Japan) with a 30-watt laser tube. A laser lens (HPDFO, Universal Laser Systems, Kanagawa, Japan) of 30 µm focal spot was used. PMMA sheets (Spiroplastic, Cairo, Egypt) of different thicknesses were used for fabrication. The laser beam was applied with 1000 pulses per inch (PPI) to develop the required design by patterning the PMMA sheets. Different laser powers and scanning speeds were applied based on the thickness of the PMMA sheet. After fabrication, the flow cell was assembled and tested twice with two different 14-mm-diameter commercial QCM sensors. The first has a fundamental frequency of 10 MHz (ALS Co., Ltd, Tokyo, Japan), and the other has 6 MHz (COLNATEC, Greenville, SC, USA). Deionized (DI) water was pumped to the surface of the sensor through the input port at different flow rates for leakage testing in both cases. The pumping process was performed using a syringe pump (NE-4000, New Era Systems, Grant, FL, USA).

### Oscillating circuit

#### Hartley oscillator working principle

**Fig. 3:**
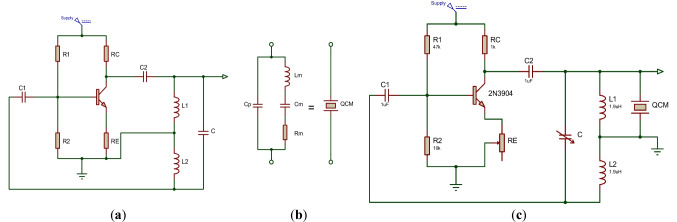
**a** Schematic diagram of the Hartley oscillator. **b** The unloaded QCM electrical model. **c** The modified QCM Hartley oscillator of the present work

The Hartley oscillator is a type of *LC* oscillator circuit that generates a sinusoidal signal at a specific frequency. It consists of an inductor, a capacitor, and a transistor. The oscillator operates on the principle of positive feedback, where a portion of the output signal is fed back to the input to sustain oscillations. The feedback is provided by a tap on the inductor coil, which divides the coil into two parts ($L1$ and $L2$). One part of the coil is connected to the collector of the transistor, and the other part is connected to the base of the transistor through a capacitor. Figure 3a illustrates a schematic diagram of the Hartley oscillator. The feedback provided by the inductor tap and capacitor causes the oscillator to oscillate at a specific frequency determined by the values of the inductor and capacitor. The frequency of oscillation can be calculated using the following equation:

1 $$f=\frac{1}{2\pi \sqrt{L*C}},$$

where $f$ is the frequency of oscillation, $L$ is the inductance of the coil ($L$ = $L1$+ $L2$), and $C$ is the capacitance of the capacitor. As can be noted from Fig. 3a, the feedback network in the Hartley oscillator is formed by the coupling capacitor ($C$ 1) and a voltage divider consisting of two resistors ($R1$ and $R2$). The voltage divider is used to bias the transistor and establish the operating point.

#### QCM equivalent circuit

The QCM sensor is electrically modeled, using the Butterworth-Van-Dyke (BVD) circuit model, as a series *RLC* arm (${L}_{m}$, ${C}_{m}$, and ${R}_{m}$) (Alassi et al. 2017). A capacitor (${C}_{P}$) added across the *RLC* arm represents the capacitance of the electrodes. Figure 3b shows the corresponding BVD electrical model of the unloaded QCM sensor. The resonance frequency of the sensor is determined based on the values of elements in the electrical model. For in-liquid applications, ${R}_{liq}$ and ${L}_{liq}$ elements are added in series with the *RLC* arm. At the same time, the added mass over the surface of the sensor is represented by a series inductance (${L}_{mass}$) (Beißner et al. 2017). Therefore, any change in the liquid or the added/removed mass will affect the oscillating frequency.

#### The modified hartley oscillator

In this work, we connect the QCM sensor in parallel with the $L1$ of the Hartley oscillator of Fig. 3a. This connection ensures the grounding of one side of the sensor, as the literature recommends (Alassi et al. 2017; Barnes 1991, 1992). The dry electrode of the QCM is connected to the ground of the circuit. This is because the dry electrode is typically used as the reference electrode, while the wet electrode is exposed to the measured sample. Connecting the dry electrode to the ground ensures that the reference electrode is at a fixed potential, allowing for accurate resonant frequency measurements. Furthermore, connecting the wet electrode to the ground may cause electrical interference or noise, which can affect the accuracy of the QCM measurements. Figure 3c presents the modified Hartley oscillating circuit used in this work.

A variable capacitor ($C$) is used to tune the oscillating circuit near the resonant frequency of the QCM sensor. This adjustment allows the use of different QCM sensors with different resonant oscillating frequencies in a specific range. A coupling capacitor ($C2$) transmits the generated AC signal to the QCM sensor and blocks any direct current (DC) coupling. A silicon NPN bipolar junction (BJT) transistor (2N3904) was used in the oscillating circuit due to its low power consumption, low cost, small size, fast switching speed, and wide availability. In addition, it is less sensitive to fluctuations in voltages and currents as compared to other BJTs. The transistor was biased, and the operating point was established using resistances of 47kΩ and 10kΩ for $R1$ and $R2$, respectively. The variable resistance ($RE$) tunes the output voltage amplitude across the QCM sensor.

The proposed oscillator was simulated using Proteus software. For testing, the oscillating circuit was first connected to a breadboard. The circuit was optimized to have a resonant frequency close to the fundamental frequency of the QCM sensor. The circuit was then transferred to a printed circuit board (PCB). The circuit was tested for different oscillating frequencies, and the range of operating frequencies has been reported. Using SMA connectors, which were mounted in the flow cell, the output of the circuit was connected to the QCM sensor. The oscillating circuit output signal was monitored using an oscilloscope (DSO5012A, Agilent Technologies, Petaling Jaya, Selangor, Malaysia).

#### Oscillating frequency measurement

The oscillating frequency of the output signal was measured using a benchtop frequency counter (53220A, Keysight, Petaling Jaya, Selangor, Malaysia) through an SMA cable. An ESP32 microcomputer (ESP32-WROOM-32, Espressif Systems, Shanghai, China) was used to measure the frequency of the oscillator using the built-in frequency counter circuit for portable mode measurements. The ESP32 was selected because of the high clock frequency that reaches 240 MHz. Using the onboard Wi-Fi capabilities of the ESP32, the measurements are then sent to a server for further data analysis.

### Pumping system

The sample solution is delivered to the QCM sensor through the tubing system of the flow cell described previously. In the current system, the flow cell is connected to a syringe pump in the benchtop mode or an integrated pumping system in the portable mode. The built-in pumping system consists of a miniature piezoelectric diaphragm micropump (SDMP306D, Takasago Electric, INC., Nagoya, Aichi, Japan) and an Arduino board for flow rate control. The piezoelectric micropump is a pump that uses the piezoelectric effect to generate pressure and move fluids. The outer dimensions of the micropump are 25 × 25 × 8.2 mm. The enabled terminal of the built-in driver of the micropump is used to program it to deliver a constant flow rate. By turning the pump on and off at a certain frequency, a pulsed flow is achieved and controlled by adjusting the duty cycle (the percentage of time the pump is on). As a first approximation, a linear relationship between the duty cycle and flow rate is assumed for the desired flow rate parameters calculations. The actual flow rate parameters, which depend on the specific characteristics of the system, such as tubing dimensions and solution properties, are then calculated through calibration.

**Fig. 4:**
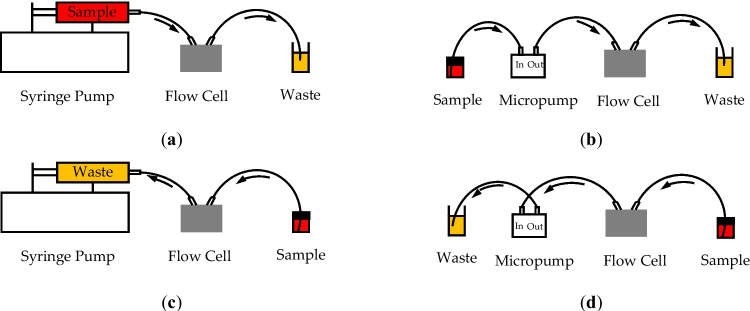
Possible flow paths in the developed system. Push configuration using: **a** syringe pump and **b** micropump. Pull configuration using: **c** syringe pump and **d** micropump. Arrows illustrate the flow direction

The developed system has two tube fittings to work as inlet and outlet ports for sample solutions. In the portable mode, the inlet port of the system is connected to the inlet of the micropump, while the output port of the system is connected to the output port of the flow cell. The output of the micropump is connected to the inlet port of the flow cell to close the loop. In the benchtop mode, the inlet and outlet ports of the flow cell are connected directly to the inlet and outlet ports of the system, respectively. One of the ports of the system is connected to a syringe fixed on the syringe pump, and the other is to a reservoir. A sample holder is designed to hold four 10-mL glass reservoirs. The holder is fixed in the system from the outside to allow changing samples without affecting the regulated temperature inside the box. There are two possible solution flow paths in the developed system. The first is to push the sample by the pump, passing through the flow cell and ending in the waste reservoir. The second is to pull the sample that passes firstly through the flow cell, then through the pump. The push and pull configurations are illustrated in Fig. 4.

### Air conditioning system

The temperature of the QCM sensor should be regulated to maintain the accuracy and reliability of the system measurements. In addition, the other oscillating circuit components, especially the capacitors, should be thermally stable (Koçum et al. 2009). In this work, the temperature inside the box was controlled using a Peltier-based air conditioner to ensure the thermal stability of all system components. Using the developed system in bioapplications performed at near room temperature eliminates the need for two separate temperature controllers. The designed air conditioning system regulates the temperature of the oscillating circuit and the flow cell containing the QCM sensor. Generally, two separate Peltier elements may be used; one for heating and the other for cooling. A single Peltier module was used in the current work to build the integrated temperature control system, reduce costs, and miniaturize the system.

**Fig. 5:**
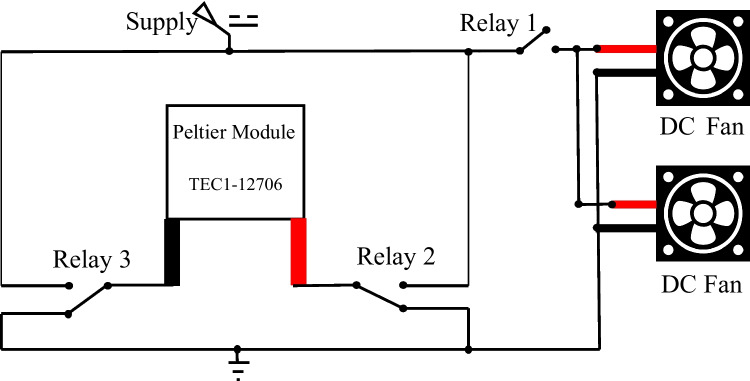
Schematic diagram of the temperature control circuit

A Peltier module is a thermoelectric device used for heating or cooling. The working side of a Peltier element can work as a cooler or heater based on the current flow direction between the two terminals of the Peltier (Mannella et al. 2014; Riffat and Ma 2003). This ability allows using a single Peltier module for heating and cooling to regulate the temperature inside the box. In this work, a Peltier module (TEC1-12706) of 40 × 40 × 3.8 mm dimensions was sandwiched between two heat sinks. The performance specifications of the Peltier module are presented in Table 1. Heat sinks were used to dissipate the power generated by the Peltier device. Two DC fans were fixed on the heat sinks to fasten the power transfer inside the box and help the power dissipation on the outer side. The temperature controller was built with an Arduino board and three relay modules, in addition to a temperature sensor (DS18B20) of -50 to 150 °C measurement range for feedback signals. A schematic diagram of the temperature control circuit is demonstrated in Fig. 5.

**Table 1:** Performance specifications of TEC1-12706 Peltier module

**Specification**	**Range**	
**Hot Side Temperature (ºC)**	25	50
**Q**_**max**_ **(W)**	50	57
**∆T**_**max**_ **(ºC)**	66	75
**I**_**max**_ **(A)**	6.4	6.4
**V**_**max**_ **(V)**	14.4	16.4
**Resistance (Ω)**	1.98	2.3

The first relay (Relay 1) controls the DC fans, while the others (Relay 2 and Relay 3) control the current flow direction between the terminals of the Peltier module. The temperature sensor is placed inside the slot of the lower cover of the flow cell, and the Arduino board controls the relays based on the temperature readings. When the temperature inside the box is higher than the set temperature, the controller sets the operating mode of the air conditioner as a cooler. On the other hand, the operating mode is set as a heater if the measured temperature is lower than the set temperature. Relay 2 is activated in the cooler mode, and relay 3 is terminated, allowing the current to flow in a specific direction. For altering the current flow to operate the heater mode, relay 2 is terminated, and relay 3 is activated. Relay 1 is activated in both modes to operate the DC fans to increase the power transfer rate and enhance the cooling and heating efficiency.

### System interface and validation

After the fabrication of each sub-system, the components were interfaced and assembled in a standard project box. A plastic box of 260 × 185 × 80 mm dimensions has been used to ensure the thermal isolation and low weight of the portable system. The QCM was mounted in the flow cell and connected to the oscillating circuit using SMA cables. The pumping system was connected to the flow cell using 1.6 mm-inner diameter tubes. The sensitivity to mass change, in terms of viscosity change, over the surface of the QCM sensor experiment was performed to verify the ability of the developed system to be used in bioapplications like the detection of intracellular bacteria in biological samples. A commercial 10 MHz QCM sensor has been used for the test.

#### Sample preparation

A series of glycerol samples with different concentrations were prepared for experimental analysis. DI water was used as a buffer zone during preparing the samples. The viscosity of both the DI water and the prepared samples was measured via a rheometer (KNX2212, KINEXUS, Malvern Panalytical Ltd, Malvern, Worcestershire, UK). Samples were prepared in 10 mL-glass reservoirs mounted on the sample holder to perform the test.

#### Experimental test

**Fig. 6:**
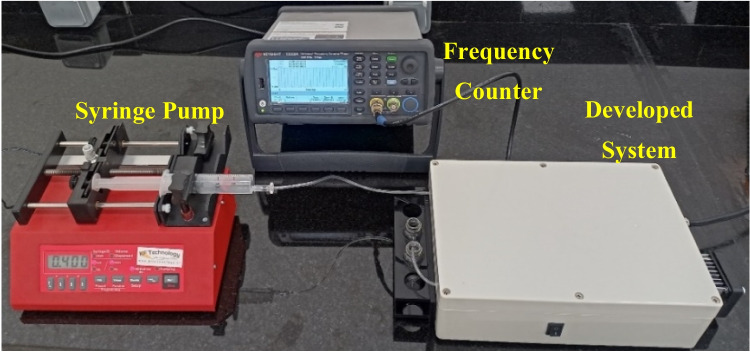
The setup of the developed system in benchtop mode

The flow rate of the liquid over the surface of the QCM sensor can affect the results of the performed test. Therefore, the optimal flow rate for this specific test was defined. Before starting the sensitivity test, a 70% alcohol solution was delivered to the sensor surface by the pumping system to clean the surface and remove any contaminants or residues. Then, DI water was pumped to the surface of the sensor, and the frequency response was monitored using the frequency counter. After the response was deemed stabilized, the first sample was introduced into the system until a new resonant frequency value was reached. The second sample was then introduced, and the process was repeated for all samples. All samples were pumped into the system at the optimal flow rate. The setup of the developed system for performing the sensitivity test is illustrated in Fig. 6.

## Results and discussion

As it is important to test for leakage before using the flow cell for any experiments to ensure accurate and reliable results, DI water was pumped to it with different flow rates for leakage testing. After the dynamic flow test was carried out, a static test was performed by filling the flow cell with DI water, and the inlet and outlet ports were sealed, ensuring no air was entering or leaving the cell. After sealing, the flow cell was left for an hour. For both cases, no leakage was observed during or after the test, indicating the proper working of the seal gasket used in the developed cell. The leakage tests were performed using the 14-mm-diameter 10-MHz and 6-MHz QCM sensors. This illustrates the possibility of using different commercial QCM sensors that match the design dimensions and the position of electrodes. Therefore, other 14-mm commercial QCM sensors can be seamlessly integrated into the system by adjusting the tank circuit components to match the required operating frequency range.

The oscillating circuit of the developed system was intended to be used with QCM sensors of fundamental frequencies in the range of 10 to 25 MHz. Therefore, Eq. (1) was used to calculate the values of the inductors and the capacitor of the tank circuit. The inductors, *L1* and *L2*, were chosen to have an inductance of 3.8 µH (1.9 µH each). Meanwhile, the capacitance of the capacitor, *C*, ranged from 10 to 70 pF. Table 2 presents some theoretical resonant frequency, ${f}_{r}$, values for different capacitance values.

**Table 2:** Calculated resonant frequency for different capacitor values of the oscillating circuit of Fig. 3c

${\varvec{C}}$**(pF)**	${{\varvec{f}}}_{{\varvec{r}}}$**(MHz)**
**10**	25.82
**20**	18.26
**30**	14.91
**40**	12.91
**50**	11.55
**60**	10.54
**70**	9.758

**Fig. 7:**
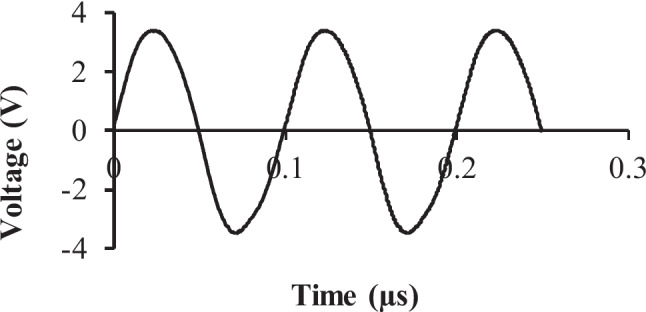
The output waveform of the oscillating circuit with a 10 MHz QCM sensor

The oscillating circuit was tested experimentally by connecting it to the 10 MHz QCM sensor and measuring the output signal using the oscilloscope. The variable capacitor was used to tune the oscillating frequency and the variable resistance to tune the amplitude of the output waveform connected across the QCM sensor electrodes. The adjusted output waveform was a sinusoidal signal with a frequency of 10 MHz, and a peak-to-peak voltage of 7 V. Fig. 7 displays the output waveform of the oscillating circuit. It is important to drive the QCM sensor with the appropriate voltage signal amplitude to avoid damaging it. Additionally, maintaining the purity of the sinusoidal signal is paramount for precise and stable frequency counting.

**Fig. 8:**
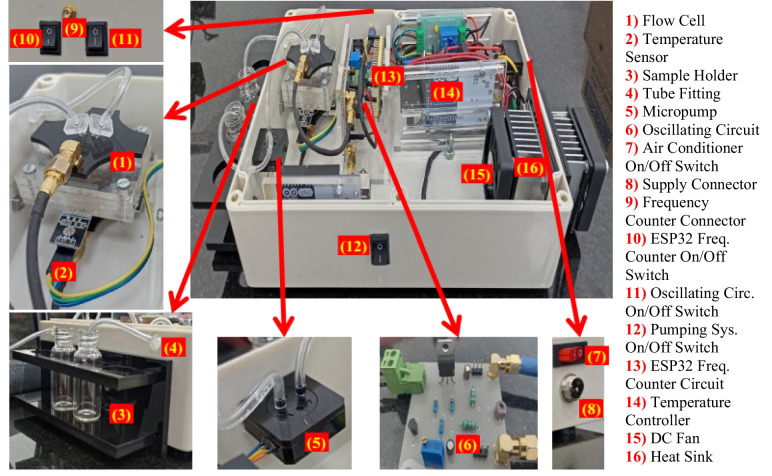
Main components of the assembled developed system

All sub-systems were assembled in the selected box, upon the conclusion of the flow cell and oscillating circuit testing. Fig. 8 shows the assembled system and its main components. The oscillating circuit and the built-in ESP32-based frequency counter were fixed near the flow cell to shorten the wiring with the QCM sensor, reducing parasitic effects. Free space in front of the inner DC fan was devoted to allowing for heat transfer, and reaching thermal equilibrium faster. The space between the micropump and the flow cell allows for integrating a microfluidic chip for further processing before or after the sensing process based on the application. The chip can also be connected to the system externally using the two external tube fittings. However, when using the portable mode in field applications, it is recommended to connect the microfluidic chip to the system from the inside to ensure the thermal stability of the measured samples. To ensure a constant flow rate and avoid sources of turbulent flow in the tubing system, tubes of the same inner diameter (1.6 mm) were used in all connections. Separate on/off controller switches were connected to each sub-system to allow controlling each individually if needed, as illustrated in Fig. 8.

The fluctuations in current flow within the Peltier module can disrupt the oscillating circuit. Hence, two separate power sources have been utilized in the developed system. The supply connector, seen in Fig. 8, is used to power the system with 12V DC via a benchtop power supply or a lithium battery when operating in portable mode. This connector powers the air conditioner and pumping systems. On the other hand, two small 9V batteries connected in series are used to power the oscillating circuit and the ESP32 frequency counter circuit. These batteries are housed inside the system box and connected to the on/off switches of both circuits. Due to their low power consumption, these batteries have a long lifespan. To ensure a stable 12 V input to the oscillating circuit, a voltage regulator IC was implemented for this purpose, as can be noted in the fabricated oscillating circuit presented in Fig. 8.

**Fig. 9:**
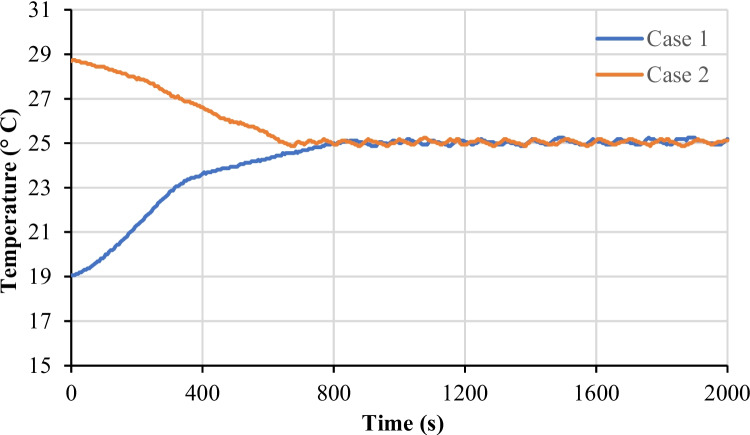
Performance of the integrated temperature controller under varying ambient conditions. The preset temperature of the system is 25 °C, and the ambient temperature is 19 °C in Case 1 and 29 °C in Case 2

The integrated temperature controller underwent testing under varying ambient conditions. In the first scenario, with an ambient temperature of 19 °C, the system achieved the preset inner temperature of 25 °C in approximately thirteen minutes. In the second scenario, operating under an ambient temperature of approximately 29 °C, the system successfully reached the set temperature in around eleven minutes. A visual representation of the recorded temperature measurements is shown in Fig. 9. The test demonstrates the capability of the temperature controller to uphold the internal temperature of the system within the specified range around the set temperature. This achievement ensures thermal stability, safeguarding the frequency measurements from being influenced by temperature fluctuations.

**Fig. 10:**
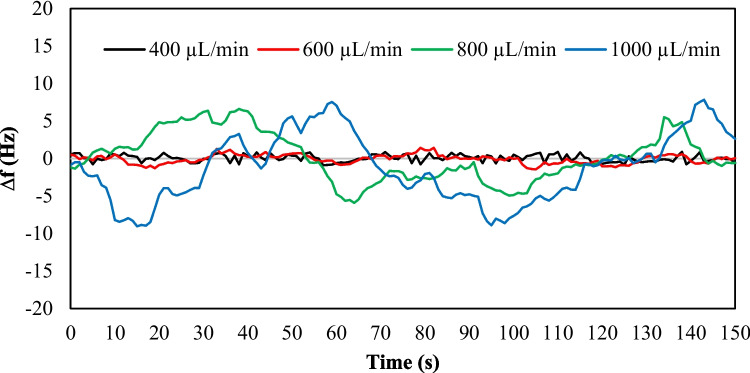
Flow rate effect on frequency measurements of the developed system

In order to test the sensitivity of the developed system to changes over the surface of the QCM sensor, a viscosity change test was conducted using a 10-MHz QCM sensor. The 10 MHz sensor was selected to match the designed operating frequency range of the oscillating circuit. Before the test, the air conditioner was turned on to maintain the box temperature at the predetermined 25 °C. This step is essential to counteract any temperature-induced effects on the frequency response, especially considering that the ambient temperature (outside the box) during the test was recorded at 20 °C. The oscillating circuit was also powered up, and the frequency was recorded until it stabilized. DI water was then introduced to the surface of the QCM sensor with varying flow rates, starting at 100 µL/min to determine the optimal flow rate. The flow rate was then increased by increments of 100 µL/min. High fluctuations were observed in the measurements due to turbulent flow at very high rates, while a very low flow rate could result in stagnant flow and an increase in test time. Fig. 10 displays the frequency response of the system at different flow rates.

Fig. 10 exhibits the measurement fluctuations increase with an increase in flow rate. The uncertainty value for the measurements presented in Fig. 10 has been calculated and is given in Table 3. The uncertainty can be calculated using the following equation:

2 $${u}_{f}=\pm \sqrt{\frac{{\sum }_{1}^{n}{\left(f-\overline{f }\right)}^{2}}{n}},$$

where ${u}_{f}$ is the uncertainty in measurements of frequency of oscillation, $\overline{f }$ is the mean of measurements, and $n$ is the total number of the measured data. These uncertainty values are specific to the current test conditions and may differ for other conditions, such as using a different solution or a different QCM sensor. The uncertainty values presented in Table 3 were used to determine the precision of the developed system and identify the optimal flow rate for the current test. A flow rate of 400 µL/min was selected as it resulted in an uncertainty of ± 0.48 Hz, which was deemed suitable for the required level of accuracy in the current test. It is noteworthy that flow rates below 400 µL/min showed no significant improvement, rendering 400 µL/min the chosen flow rate to expedite the test and optimize time efficiency. It is also important to note that the optimal flow rate may vary for different systems, and the sensitivity of the QCM sensor to flow rate changes increases with an increase in the fundamental frequency of the sensor. Due to the use of the built-in temperature controller, the measurements are recorded at a constant temperature. Therefore, no trends were observed in the measurements at a constant flow rate, as can be noted in Fig. 10. The set temperature can be easily adapted by programming the controller of the air conditioner.

**Table 3:** Uncertainty in frequency measurements of Fig. 10

**Flow Rate (µL/min)**	**Uncertainty (Hz)**
1000	± 4.52
800	± 3.26
600	± 0.60
400	± 0.48

The syringe pump was used to pump the solutions to the flow cell in a precisely controlled flow in the benchtop mode. Because a constant flow rate is necessary for accurate measurements with QCM in portable mode also, the micropump was calibrated to deliver the samples with a 400 µL/min flow rate in the current application. The controller of the pumping system can program the micropump to work at the required flow rate based on the application and the QCM sensor. This possibility of adaptation is one of the advantages of the developed system.

**Fig. 11:**
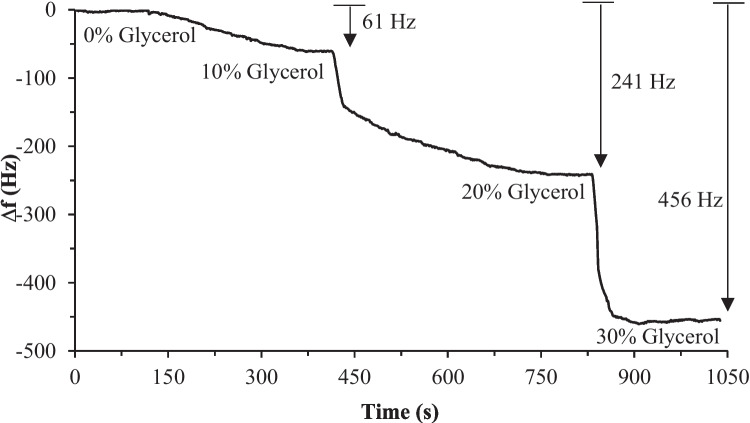
Frequency response of the QCM sensor during the sensitivity test. The preset temperature of the system is 25 °C, and the ambient temperature is 20 °C. The data points were statistically analyzed to determine the sensitivity of the system

**Fig. 12:**
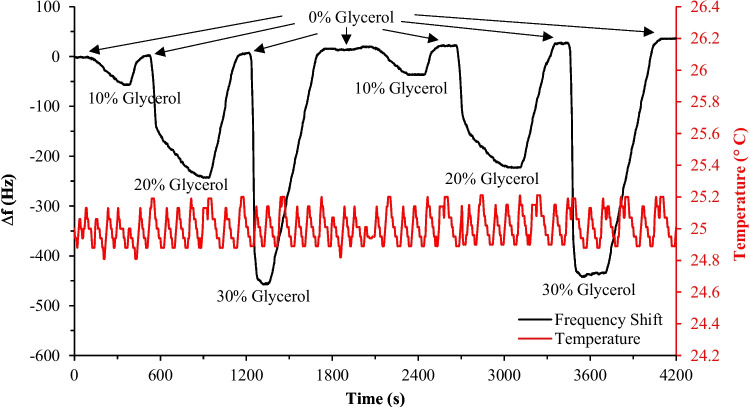
Frequency response of the QCM sensor during the test of repeatability and reversibility of the system. The preset temperature of the system is 25 °C, and the ambient temperature is 20 °C. The data points were statistically analyzed to assess the repeatability and reversibility of the system

**Fig. 13:**
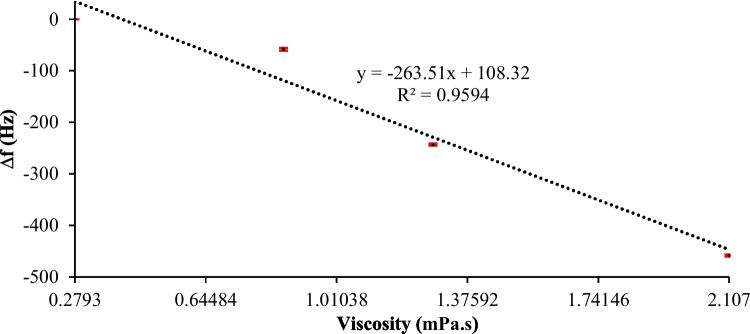
Frequency shift versus the concentration of glycerol samples using a 10 MHz QCM sensor. The linear regression analysis was performed using the data generated from Figs. 11 and 12

DI water was considered the reference fluid (0% glycerol) to perform the test, and concentrations of 10%, 20%, and 30% glycerol samples were prepared. The viscosity value of each sample at 25 °C is reported in Table 4. The frequency response of the oscillating circuit during the experimental test of injecting all samples has been monitored using the frequency counter. Fig. 11 shows the frequency shift of the circuit for each sample as a comparison to the baseline frequency of the reference fluid. As expected, the oscillating frequency decreased as glycerol concentration increased due to the viscosity increase, as noted in Fig. 11. To ensure the reliability of the system, the procedures of the first test were conducted two additional times to assess the repeatability of the system. In this second test, the reversibility of the system was also examined by introducing a 0% glycerol sample after each concentration. The response of the system to these different glycerol samples is illustrated in Fig. 12, demonstrating the repeatability and reversibility of the measurements. Despite the ambient temperature being recorded at 20 °C during the tests, the built-in temperature controller effectively maintained the preset internal temperature of the system, as indicated in Fig. 12. The data obtained from the frequency response of the system in both tests were statistically analyzed were plotted as a function of the glycerol concentration, as illustrated in Fig. 13. A linear regression analysis was performed to determine the sensitivity of the system to the glycerol concentration. The sensitivity of the system using a 10-MHz QCM sensor was found to be 263.51 Hz/mPa.s. Therefore, the system can potentially be used to detect various analytes in bioapplications, including proteins, DNA, and bacteria.

**Table 4:** Viscosities of glycerol samples with different concentrations at 25 °C, essential for the linear regression analysis of the sensitivity test

**Concentration**	**Viscosity (mPa.s)**
**0% Glycerol (DI Water)**	0.2793
**10% Glycerol**	0.8621
**20% Glycerol**	1.2800
**30% Glycerol**	2.1070

**Fig. 14:**
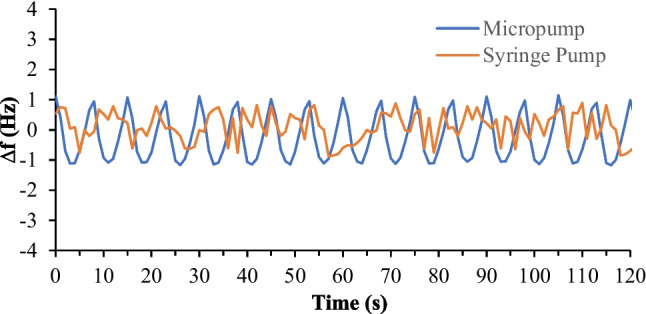
Pump type effect on frequency measurements of the developed system. Samples are delivered to the sensor surface at 400 µL/min flow rate in both cases. Measurements exhibit an average frequency shift of approximately 0 Hz in both cases

The sensitivity of the system to flow fluctuations, influenced by the pump type, underwent examination. DI water was pumped to the QCM sensor surface at a consistent flow rate of 400 µL/min using both the syringe pump and the micropump. Figure 14 illustrates the frequency response of the sensor with both pumps. Although the average measurements are comparable in both cases, a periodic frequency response appears when using the micropump, as depicted in Fig. 14. This periodic response stems from the cyclic movements of the membranes of the micropump, deemed negligible in the current application due to its minimal impact on measurement accuracy within the specified ranges. To enhance measurement stability in small-range applications, it is recommended to incorporate miniature flow stabilizers, such as a microfluidic chip, in the flow before entering the flow cell.

In addition to its portability, the developed system has the advantage of being cost-effective. The entire system, including the micropump, costs less than 200 USD, which is approximately one-fifth of the cost of the least expensive state-of-the-art commercial benchtop QCM systems. While the developed system will be used for field tests in the future, further research will focus on improving its sensitivity and measurement accuracy. Future work will also focus on the functionalization of the QCM sensor surface to enable the selective detection of specific analytes. Biomacromolecules of diagnostic importance based on host-guest interactions with binding capacity estimations will be investigated. Biomonitoring of such interactions like enzyme-substrate, antigen-antibody, ligand and receptor or DNA counterparts could help in molecular architectonics of myriad theragnostic applications. Additionally, the integration of the microfluidic chips with the QCM sensor will be explored to enable the real-time monitoring of analytes through point-of-care devices.

## Conclusions

In this work, a compact and portable QCM-based system with a total cost of less than 200 USD was designed and fabricated for use in liquid biosensing. In addition to the portable mode, the developed system can be used in benchtop mode. A new low-cost and easy-to-use flow cell design was presented and tested. The system uses a modified Hartley oscillator with 14 mm-diameter commercial QCM sensors ranging from 10 to 25 MHz fundamental frequencies. The frequency response of the system is measured using a benchtop frequency counter or an integrated ESP32-based frequency counter that sends measurements to a server through the built-in Wi-Fi module. A micropump-based pumping system to deliver the samples to the surface of the sensor was integrated into the developed system, in addition to the ability to connect the system to external pumps in benchtop mode. An integrated temperature controller was designed to keep the temperature of the system components constant to minimize the effect of temperature on the measured frequency response. All sub-systems were assembled in a small plastic box, and separate on/off controller switches were connected to each sub-system to control them individually if needed. The system was evaluated for being used in liquid biosensing by testing its sensitivity to the concentration of glycerol samples. The repeatability and reversibility of the system were evaluated. It was found to have a sensitivity of 263.51 Hz/mPa.s using a 10-MHz QCM sensor. Results suggest that the system can be used for quantitative measurements of the concentration of analytes in bioapplications. Integrating microfluidic chips through the detachable tubes in the developed pumping system will be a subject of deeper study.

## Data Availability

All data generated or analyzed during this study are included in this published article.
